# Progress in Local Treatment of Breast Cancer: A Narrative Review

**DOI:** 10.1055/s-0040-1712125

**Published:** 2020-06

**Authors:** Francisco Pimentel Cavalcante, Eduardo Camargo Millen, Felipe Pereira Zerwes, Guilherme Garcia Novita

**Affiliations:** 1 Breast Surgery Service, Hospital Geral de Fortaleza, Fortaleza, CE, Brazil; 2 Breast Surgery Service, Clínica São Vicente, Rio de Janeiro, RJ, Brazil; 3 School of Medicine, Breast Surgery Service, Pontifícia Universidade Católica do Rio Grande do Sul, Porto Alegre, RS, Brazil; 4 Breast Surgery Service, Grupo Américas, São Paulo, SP, Brazil

**Keywords:** breast cancer therapy, breast-conserving therapy, sentinel node biopsy, mastectomy, neoadjuvant and adjuvant therapy, terapia de câncer de mama, terapia de conservação da mama, biópsia de linfonodo sentinela, mastectomia, terapia neoadjuvante e adjuvante

## Abstract

The present paper reports on the local treatment of breast cancer from a historical perspective. A search for articles written in English was made in the Medline and EMBASE databases, and 40 papers were selected. Over the past 10 years, various randomized, controlled clinical trials on the local treatment of breast cancer indicated that patients with the same molecular subtype may receive different individualized surgical treatments aimed at optimizing systemic adjuvant therapy. With a view to retaining the gains made in disease-free and overall survival, surgical techniques have advanced from radical surgery to conservative mastectomies, thus reducing sequelae, while adjuvant and neoadjuvant therapies have contributed toward controlling the disease, both distant metastases and local recurrence. Current studies evaluate whether future breast cancer therapy may even succeed in eliminating surgery to the breast and axilla altogether.

## Resumo

Este artigo discute o tratamento local do câncer de mama a partir de uma perspectiva histórica. Uma busca de artigos publicados em inglês foi realizada nas bases de dados Medline e EMBASE, sendo selecionados 40 artigos. Nos últimos 10 anos, vários ensaios clínicos controlados e randomizados sobre o tratamento local do câncer de mama indicaram que pacientes com o mesmo subtipo molecular podem receber diferentes tratamentos cirúrgicos individualizados com o objetivo de otimizar a terapia adjuvante sistêmica. Pretendendo reter os ganhos obtidos na sobrevida livre de doença e na sobrevida global, as técnicas cirúrgicas avançaram progressivamente da cirurgia radical para mastectomias conservadoras, reduzindo sequelas, enquanto as terapias adjuvantes e neoadjuvantes contribuíram para o controle da doença, tanto em relação às metástases distantes quanto à recorrência local. Estudos atuais avaliam se a terapia futura contra o câncer de mama poderá até mesmo eliminar a cirurgia da mama e da axila por completo.

## Introduction

Dramatic progress has been made in the local treatment of breast cancer in recent years. Surgical techniques have advanced from radical surgery to breast-conserving surgery and to even more conservative mastectomies. It is no longer possible to justify severe sequelae in women treated for breast cancer. In parallel, adjuvant and neoadjuvant therapies have allowed control of the disease, not only with respect to distant metastases, but also local recurrence. The purpose of the present article is to review the progress that has been made in the local treatment of breast cancer up to the present time from a historical perspective.

## Methods

A search was made of the MEDLINE and EMBASE databases using the medical subject headings: breast cancer therapy, breast-conserving therapy, sentinel node biopsy, mastectomy, neoadjuvant chemotherapy, and adjuvant therapy. Papers not published in English and case reports were excluded. The authors reviewed 1,077 abstracts from meta-analyses, randomized clinical trials, and cohort, longitudinal or prospective studies reporting on oncologic outcomes following breast cancer treatment. Randomized clinical trials were the preferred choice, except in situations in which no such studies existed (Table 1). Studies with at least 2 years of follow-up were preferred. Most of the identified studies were excluded because of their retrospective review design. The authors selected the following topics to create an appropriate chronological narrative: radical mastectomy, the era of clinical trials, conservative breast surgery, conservative mastectomy, the role of radiotherapy in breast surgery, the axillae, and the future. The studies on each topic were evaluated separately. The authors decided that a classic article by Halsted published in 1907^1^ would serve as the starting point for this narrative. Articles written in English were retrieved and read in full by at least two of the authors. Forty papers were selected for use in the present review, including the article published in 1907 and the more recent literature up to May 2019. No classification system was used to rate the level of evidence.^2^ ^3^ ^4^ ^5^ ^6^ ^7^ ^8^ ^9^ ^10^ ^11^ ^12^ ^13^ ^14^ ^15^ ^16^ ^17^ ^18^ ^19^ ^20^ ^21^ ^22^ ^23^ ^24^ ^25^ ^26^ ^27^ ^28^ ^29^ ^30^ ^31^ ^32^ ^33^ ^34^ ^35^

**Table 1:** Characteristics of the randomized clinical trials included in the review

Study	Study period	Follow-Up	Intervention	Total sample size
Fisher et al^3^	1971–1974	25 years	Radical mastectomy Total mastectomy Total mastectomy + radiotherapy	1,079
Fisher et al^5^	1976–1984	20 years	Total mastectomy Lumpectomy Lumpectomy + radiotherapy	1,851
Veronesi el al^6^	1973–1980	20 years	Radical mastectomy Quadrantectomy + radiotherapy	701
van Dongen et al^7^	1980–1986	10 years	Modified mastectomy Lumpectomy + radiotherapy	868
Poggi et al^8^	1979–1987	18.4 years	Modified mastectomy Lumpectomy + radiotherapy	237
Blichert-Toft et al^9^	1983–1989	6 years	Mastectomy Lumpectomy + radiotherapy	905
Arriagada et al^10^	1972–1979	15 years	Mastectomy Conservative surgery + radiotherapy	179
Fisher et al^11^	1982–1988	10 years	Tamoxifen Placebo	2,818
Fisher et al^12^ NSABP 13 NSABP 19		8 years	NSABP 13: Methotrexate + fluorouracil Placebo NSABP 19: Cyclophosphamide + methotrexate + fluorouracil Methotrexate + fluorouracil	760 1,095
Haviland et al^19^ START A START B	1999–2002	10 years	START A: 25 radiotherapy fractions (50Gy) 13 radiotherapy fractions (41.6 or 39 Gy) START B: 25 radiotherapy fractions (50 Gy) 15 radiotherapy fractions (40 Gy)	2,236 2,215
Hughes et al^20^	1994–1999	10 years	≥ 70 years: Lumpectomy Lumpectomy + radiotherapy	636
Kunkler et al^21^	2003–2009	5 years	≥ 65 years: Lumpectomy Lumpectomy + radiotherapy	1,326
Krag et al^24^	1999–2004	8 years	Negative sentinel lymph node: Sentinel lymph node alone Sentinel lymph node + axillary lymph node dissection	5,611
Giuliano et al^25^	1999–2004	6.3 years	Positive sentinel lymph node (1 or 2): Sentinel lymph node alone Sentinel lymph node + axillary lymph node dissection	891
Donker et al^26^	2001–2010	5 years	Positive sentinel lymph node: Axillary radiotherapy Sentinel lymph node + axillary lymph node dissection	1,425
Sávolt et al^27^	2002–2009	5 years	Positive sentinel lymph node: Axillary radiotherapy Sentinel lymph node + axillary lymph node dissection	526
Galimberti et al^28^	2001–2010	5 years	Positive sentinel lymph node (micrometastases): Sentinel lymph node alone Sentinel lymph node + axillary lymph node dissection	934
Solá et al^29^	2001–2008	5 years	Positive sentinel lymph node (micrometastases): Sentinel lymph node alone Sentinel lymph node + axillary lymph node dissection	247
Masuda et al^34^	2007–2012	5 years	HER2-negative invasive residual disease: Capecitabine Placebo	910
von Minckwitz et al^35^	2013–2015	3 years	HER2-positive invasive residual disease: Trastuzumab T-DM1	1,486

Abbreviations: HER2, human epidermal growth factor receptor 2; NSABP, National Surgical Adjuvant Breast and Bowel Project; T-DM1, trastuzumab emtansine.

### Radical Mastectomy

William Stewart Halsted first coined the idea of radical breast surgery at the end of the 19^th^ century. Because most recurrences occurred close to the site of resection, Halsted^1^ hypothesized that the margins were probably inadequate and that wide and complete resection of the organ in a block that included the internal mammary nodes and pectoral muscles would be necessary to ensure local control and cure of the disease. He postulated the centrifugal theory of spread, in which the disease would spread in an organized manner from a focus in the breast, initially to the axillary lymph nodes and then to distant organs. Radical mastectomy was effective in reducing the rates of local recurrence; however, significant morbidities, such as natural anterior mobilization of the shoulder following surgery and clinically significant lymphedema, were common. Furthermore, almost half the patients operated died from distant recurrence in the initial years. Halsted^1^ proposed increasing the surgery site to include up to the supraclavicular fossa and cervical region. In a study presented at the beginning of the 20^th^ century, of 40 women with cervical lymph node involvement, only three survived. These findings raised some questions: *Does radical mastectomy alter the chance of survival in the case of advanced tumors?* and *Did “cured” women with a more initial stage of the disease need such extensive surgery?* These issues were already being discussed at that time; however, radical surgery would remain as standard for many years to come before it could be tested in a clinical trial.

### The Era of Clinical Trials

Halsted's centrifugal theory and radical mastectomy remained unchallenged for several decades. Questioning the effectiveness of radical treatment, George Crile^2^ proposed a less extensive surgery referred to as *simple mastectomy*. Finally, in the 1970s, Bernard Fisher,^3^ who was driven by the “systemic disease” hypothesis and by the feminist politics of the era, conducted the National Surgical Adjuvant Breast and Bowel Project (NSABP) B-04 multicenter trial, with sufficient statistical power to compare radical mastectomy with simple mastectomy according to the axillary status (clinically negative or positive). Women with clinically negative axillae (*n* = 1,079) were randomized to standard radical surgery, simple mastectomy (without axillary dissection), or simple mastectomy (without axillary dissection) together with radiotherapy. In 2002, after 25 years of follow-up, no significant difference in distant metastasis and overall survival was found. While in the group submitted to radical surgery, around 40% of the patients presented with lymph node metastasis following axillary dissection, in the group submitted to simple mastectomy without dissection and without radiotherapy, less than half of the cases presented with visible axillary disease during follow-up. These results had an irreversible impact on local management (breast and axilla) but also opened the doors to adjuvant treatment for breast cancer in the following decades.

### Conservative Breast Surgery

Historically, conservative surgery came on the heels of the NSABP B-04 trial.^3^ Nevertheless, the idea was not innovative. In London, Geoffrey Keynes,^4^ relatively unknown in the history of breast cancer treatment, was already testing lumpectomy together with radiation in the 1920s and reporting rates of control of the disease that were similar to those found with radical surgery. The technique did not become popular at that time, mainly because of the fame of Halsted's radical mastectomy. Beginning in the 1970s, 6 prospective randomized studies were conducted to test Keynes' hypothesis, the best known being the American NSABP B-06 and the Milan study, which were conducted, respectively, by Fisher and Veronosi.^5^ ^6^ ^7^ ^8^ ^9^ ^10^ However, while the Italian study recommended resection of the tumor together with adjacent normal tissue and the associated skin island, the NSABP B-06 study advocated resection of the lesion together with sufficient normal tissue to ensure a good cosmetic outcome without obligatorily removing the skin over the tumor. At the time, the oncological safety involved in the two procedures was heatedly debated. The American study randomized a total of 2,163 women with initial stage breast cancer, tumors of up to 4 cm and negative or positive axillae to undergo radical mastectomy, lumpectomy with radiotherapy, or lumpectomy alone. After 20 years of follow-up, the rate of ipsilateral local recurrence in the group submitted to lumpectomy with radiotherapy was 14.3%. In the Italian study, women with tumors of up to 2 cm and clinically negative axillae were submitted to radical mastectomy or quadrantectomy with radiotherapy. After 20 years of follow-up, 8.8% of women in the group submitted to conservative surgery had local recurrence of the disease compared with 2.3% in the group submitted to mastectomy. Currently, conservative surgery without removal of the skin (lumpectomy or segmental mastectomy) is the form of breast-conserving surgery most widely used worldwide. Studies on breast-conserving surgery have found no differences in survival when this technique is compared with mastectomy; however, local recurrence rates were a concern, since they were still considered high. For many years, the debate focused on the minimum amount of normal tissue (margins) that had to be resected to minimize these recurrences. Of those pioneering studies, only the NSABP B-06 recommended establishing free margins, with the minimal margin required being defined as “no ink on tumor.” With the introduction of mammographic screening, reduction in the initial tumor load, and improvements in pathology protocols, but, especially, with the advent of systemic treatment, the incidence of local recurrences has currently dropped. The use of hormone therapy in the NSABP B-14 study reduced the rate of local recurrence from 14.7% (placebo) to 4.3% (tamoxifen group).^11^ Likewise, in the NSABP B-13 trial, the use of chemotherapy reduced the local recurrence rate from 13.4 to 2.6%.^12^ The current aim of breast-conserving surgery with multimodal treatment is not to merely sterilize the foci of the disease in the breast, but also to attack undetectable foci outside the index tumor, thus confronting an old problem in breast surgery. Even with the paradoxical reduction in the recurrence rate with less extensive surgery, the size of the margin remains a controversial issue. Many patients continued to be submitted to margin expansion, even when margins were already clear. More recently, based on a meta-analysis, the American Societies of Surgical Oncology, Radiotherapy and Clinical Oncology issued a consensus recommendation that the minimum margin required should be one of “no ink on tumor.”^13^ In summary, that study compared the margin in breast-conserving surgery for initial stage breast cancer using two models. The first model compared negative margins with positive margins, resulting in a significant difference favoring negative margins insofar as local control of the disease was concerned. In the second model, clear margins were compared with wider margins (established references of 1, 2, and 5 mm), with similar results being found, leading to the conclusion that wider margins are unnecessary. The study did not evaluate patients who had undergone systemic neoadjuvant therapy; however, this does not necessarily mean that wider margins are required in patients not receiving adjuvant systemic therapy. Moreover, the consensus panel does not rule out the possibility of wider margins when clinically necessary, as in cases in which residual disease is identified through imaging tests, for example, thus warranting re-excision. Traditionally used on inoperable tumors since the 1970s, neoadjuvant chemotherapy began to be used also on operable tumors to facilitate the surgical procedure, thus increasing the rates of breast-conserving surgery. Systemic therapy may also attack possible micrometastases in the body before surgery. A great debate over the years was whether, in conservative surgery, resection should be limited to the residual area or whether it should be extended to the entire previous extent of the disease prior to neoadjuvant chemotherapy. A recent meta-analysis covering 1983 to 2002 involved 10 randomized studies with 4,756 women and compared neoadjuvant chemotherapy with the same treatment given as adjuvant therapy.^14^ After a mean follow-up time of 15 years, neoadjuvant chemotherapy was associated with a greater rate of local recurrence: 21.4% for neoadjuvant chemotherapy versus 15.4% for adjuvant chemotherapy, an absolute increase of 5.5% with no effect on distant recurrence or mortality. In fact, two of these studies involved patients who responded so well to treatment that they were not submitted to surgery after neoadjuvant chemotherapy, resulting in higher recurrence rates. When these cases were removed from the analysis, the absolute difference in recurrence dropped to 3.2%. Nonetheless, this was a meta-analysis of older studies that preceded current knowledge on molecular subtypes and human epidermal growth factor receptor 2 (HER2) status; hence, without the benefit of anti-HER2 therapy. Many of these patients were not submitted to analysis of their hormone receptor status. Likewise, most of the women did not use taxanes, while some were treated with cyclophosphamide, methotrexate and 5-fluorouracil chemotherapy; however, the great majority used a regimen of anthracyclines. The clinical evaluation of these women consisted basically of physical examination and mammography. Factors such as the current use of radiology, including preoperative marking, which is associated with better pathology features, as well as the use of better systemic treatments and, above all, better individualization according to molecular subtype allow greater safety in breast-conserving surgery following neoadjuvant chemotherapy.

### Conservative Mastectomies

Many patients will undergo mastectomy. Skin-sparing mastectomy and skin-sparing mastectomy with preservation of the nipple-areola complex have become popular. The possibility of preserving all the skin makes breast reconstruction easier and minimizes scarring; however, no randomized controlled trials have been conducted to compare conventional mastectomy or even breast-conserving surgery with these types of mastectomy. A meta-analysis of observational studies with over 3,739 patients compared skin-sparing mastectomy with standard mastectomy and found no differences in the rate of local recurrence between the two: 4.0% versus 6.2% for skin-sparing mastectomy.^15^ Indeed, skin-sparing mastectomy has become standard. Conversely, skin-sparing mastectomy with preservation of the nipple-areola complex is more controversial, since, unlike skin-sparing mastectomy, in which there is a dissection plane of fatty tissue between the skin and the breast parenchyma, there is no clear plane behind the nipple-areola complex, which means that a certain amount of breast tissue has to be left. A retrospective analysis conducted in Italy assembles the best evidence on nipple-sparing mastectomy.^16^ It involved 1,989 patients who were submitted to the procedure, 1,711 with invasive carcinoma and 278 with ductal carcinoma in situ. After 94 months of follow-up, the local recurrence rate was 5.3% in the invasive carcinoma group and 4% in the ductal carcinoma in situ group. Only 6.7% of the patients with invasive carcinoma (with axillary metastases in half of these cases) underwent comprehensive radiotherapy of the breast (78% had intraoperative radiotherapy in the nipple-areola complex, and 15% had no radiotherapy at all). Recently, the choice of incision in nipple-sparing mastectomy has been debated, since reconstruction and satisfactory esthetic outcome depend on how the mastectomy is performed. Incisions in the upper outer quadrant facilitate the approach but leave a scar that may be apparent and as stigmatizing as radical surgery, while inframammary incisions are more discrete, but are not appropriate for all breasts. The periareolar option may represent a middle ground, but these incisions have historically been associated with greater necrosis of the nipple-areola complex. However, in a recent analysis, our group found low rates of necrosis of the nipple-areolar complex (9.6%; with 3.2% being cases of total necrosis) and concluded that the periareolar approach can be used.^17^

### The role of Radiotherapy in Breast Surgery

Comprehensive breast radiotherapy is a prerequisite for the viability of conservative surgery. Some studies have evaluated the role of radiotherapy in controlling the disease. In the NSABP B-06 trial, radiotherapy associated with lumpectomy reduced the rate of ipsilateral recurrence to 14.3% compared with 39.2% in the no radiotherapy group, irrespective of lymph node status.^5^ A meta-analysis of 17 randomized studies involving 10,801 women compared radiotherapy versus no radiotherapy after conservative surgery.^18^ Cancer recurrence was reduced by half when radiotherapy was given, with an effect on survival in ⅙. Overall, radiotherapy reduced the 10-year rate of any recurrence (local or distant) from 35 to 19.3%, an absolute difference of 15.7% (95%CI: 13.7–17.7%; *p* < 0.00001). Therefore, not providing radiotherapy in breast-conserving surgery should be the exception. Currently, not only is standard fractionated radiotherapy available, but also hypofractionated radiotherapy can be safely performed in selected cases. The UK Standardisation of Breast Radiotherapy (START) trial compared the standard regimen of 50 Gy in 25 fractions with hypofractionation (40 and 41.6 Gy, respectively, in 15 or 13 fractions) in patients with early breast cancer (pT1–3a pN0–1 M0) submitted to conservative surgery, and found no differences with respect to control of the disease or tolerability.^19^ More recently, two controlled studies evaluated the possibility of excluding radiotherapy in elderly patients with initial stage tumors. The first was the Cancer and Leukemia Group B (CALGB) 9343, which included patients of 70 years of age or more with hormone receptor-positive tumors, T1N0 (HER2 was not excluded, since randomization occurred between 1994 and 1999), comparing surgery plus radiotherapy with surgery alone.^20^ Over a 10-year follow-up period, the local recurrence rate was 2% in the radiotherapy group and 10% in the group submitted to surgery alone, with survival and mastectomy rates being similar in both groups. The second study was the PRIME II (Postoperative Radiotherapy in Minimum-Risk Elderly) trial, conducted in the United Kingdom with patients over 65 years of age and tumors smaller than 3 cm (HER2 not evaluable).^21^ Over a 5-year follow-up period, local recurrence was 1.3% in the group without radiotherapy compared with 4.1% in the group submitted to radiotherapy, findings that are in agreement with the first report on the CALGB 9343 study. Radiotherapy also plays an important role following mastectomy. In general, as a concept, indications should be similar in radical surgery and in breast-conserving mastectomy. Traditionally, radiotherapy is indicated for patients with 4 or more affected lymph nodes, positive surgical margins or in the case of tumors over 5 cm. There is debate regarding the role of radiotherapy in patients with 1–3 metastatic lymph nodes. A meta-analysis that included 8,135 women in 22 trials submitted either to radiotherapy following mastectomy or to surgery alone showed that in patients with 1–3 positive lymph nodes, radiotherapy reduced locoregional recurrence in 10 years (0.68; 95%CI: 0.57–0.82) and mortality in 20 years (0.80; 95%CI: 0.67–0.95).^22^ Radiotherapy had no effect in patients with negative lymph nodes in that study. The role of secondary factors such as age, molecular subtype, and angiolymphatic invasion in the decision regarding whether to use radiotherapy following mastectomy remains controversial, with no universal consensus. Also, the role of radiotherapy in the internal mammary nodes has been debated, particularly following the publication of the results of the European Organisation for Research and Treatment of Cancer (EORTC) 22922 and NCIC (National Cancer Institute of Canada) MA.20 studies.^23^ Questions regarding which patients need regional nodal irradiation therapy need to be answered in the future.

#### Axillae

Axillary lymph node dissection has been standard practice in the assessment of the axilla since the time of Halsted,^1^ irrespective of clinical nodal status. The NSABP B-04 trial raised important questions on the role of axillary dissection in breast cancer treatment, since less than half the patients with possible lymph node involvement presented with axillary recurrence in the group not submitted to axillary dissection.^3^ Nevertheless, even after that study, dissection continued to be standard, because it provided excellent local control and also provided the grounds for deciding on adjuvant treatment, either radiotherapy or chemotherapy. It was only in the 1990s that lymphatic drainage and the concept of the sentinel lymph node biopsy were understood. Subsequently, the NSABP B-32 trial, in which 5,000 women with clinically negative axillae underwent sentinel lymph node mapping, resulted in an identification rate of 97.2%.^24^ Those patients with a negative sentinel node were then randomized to axillary dissection or expectant management. After 10 years of follow-up, there was no significant difference between the groups regarding axillary recurrence and overall survival despite the false-negative rate of around 9.8% (17.7% when only 1 sentinel lymph node was identified). More women in the group submitted to axillary dissection underwent chemotherapy, suggesting an influence on treatment plan at that time, since some of these patients would have had positive axillae at the definitive biopsy. The NSABP B-32 trial also evaluated the role of occult node metastases.^24^ Here, 15.9% were identified in women with negative sentinel lymph nodes, with 2/3 consisting of isolated cells in the lymph node. Occult metastases were prognostic, although the absolute difference was small (1.2% overall survival and 2.8% disease-free survival).

The advent of sentinel lymph node mapping was a major milestone in the history of breast cancer surgery, resulting in a significant reduction in the rates of lymphedema. Nevertheless, the majority of patients with positive sentinel lymph nodes had no additional metastases. The American College of Surgeons Oncology Group (ACOSOG) Z0011 trial was conducted to evaluate the role of axillary dissection in positive sentinel lymph nodes in patients with clinically negative axillae prior to surgery.^25^ Although the initial results of that study were presented in 2010, randomization began in 1999 and ended in 2004, still at the beginning of the sentinel lymph node era and also before molecular subtypes were understood (HER2 measurement in initial stage breast cancer became available only after its randomization procedure). The Z011 study was a non-inferiority trial that included patients with positive sentinel lymph nodes and compared sentinel lymph node dissection plus axillary dissection versus sentinel lymph node dissection alone without specific axillary treatment. The study was terminated early due to poor recruitment, and with fewer events than previously programmed. The patients were submitted to breast-conserving surgery and should have received full breast radiotherapy and systemic therapy (hormone therapy and/or chemotherapy). The limit was established as two positive sentinel lymph nodes, with no minimum number of resected lymph nodes. After a mean follow-up time of 6.3 years, there was no statistically significant difference between the groups in terms of axillary recurrence or overall survival.

Among the limitations of that study, there was a breach of protocol in some cases, since some patients received high tangential fields in breast radiotherapy (the same in the two groups); however, on the other hand, around 10% of the women had no radiotherapy at all, favoring the non-experimental arm. The selection of patients with more favorable biology was also controversial. However, since HER2 status was not requested in initial stage breast cancer at that time, there was no way of selecting the subtype (the number of HER2-positive patients in the study was unknown and they were not treated with anti-HER2 therapy). The short follow-up time was another topic of debate; however, the scenario did not change over 10 years of follow-up. Finally, the statistical power of the study represented a problem, since the number of patients included was small and there were few events. For this final reason, it is highly improbable that there would be any change in the results. Furthermore, the Z0011 study is no longer alone, since four randomized studies were presented in sequence.^26^ ^27^ ^28^ ^29^ The After Mapping of the Axilla: Radiotherapy Or Surgery (AMAROS) and Optimal Treatment Of the Axilla - Surgery Or Radiotherapy (OTOASOR) studies compared axillary dissection with axillary radiotherapy and, although residual axillary disease was found in around 33% and 38% of cases, respectively, there was no significant difference in terms of axillary recurrence or overall survival.^26^ ^27^ The Italian study International Breast Cancer Study Group (IBCSG) 23–01 and the Spanish study AATRM (Agència DÁvaluació de Tecnologia i Recerca Mèdiques de Catalunya) 048/13/2000 reported similar clinical outcomes, with 13% of residual axillary disease; however, only patients with micrometastases were included (Table 2).

**Table 2:** Residual axillary disease following sentinel lymph node mapping, lymph node recurrence, and strategies used as alternatives to axillary lymph node dissection, either in cases of node-negative (study NSABP B-32 only) or node-positive patients

Study	Residual disease	Sentinel lymph node status	Lymph node recurrence	Alternatives to axillary dissection
NSABP B-32^24^	10%*	Negative	0.7 (8 years)	Sentinel lymph node biopsy
ACOSOG Z0011^25^	27%	Positive	1.5% (10 years)	Sentinel lymph node biopsy**
IBCSG 23–01^28^	13%	Positive	2.0% (9.7 years)	Sentinel lymph node biopsy
AATRM 048/13/2000^29^	13%	Positive	2.5% (5 years)	Sentinel lymph node biopsy
AMAROS^26^	33%	Positive	1.1% (5 years)	Sentinel lymph node biopsy and radiotherapy
OTOASOR^27^	38%	Positive	1.7% (8 years)	Sentinel lymph node biopsy and radiotherapy

Abbreviations: AATRM, Agència DÁvaluació de Tecnologia i Recerca Mèdiques de Catalunya; ACOSOG, American College of Surgeons Oncology Group; AMAROS, After Mapping of the Axilla: Radiotherapy Or Surgery; IBCSG, International Breast Cancer Study Group; NSABP, National Surgical Adjuvant Breast and Bowel Project; OTOASOR, Optimal Treatment Of the Axilla - Surgery Or Radiotherapy.

* Residual disease estimated from false-negative rates.

** Some patients in the study were submitted to radiotherapy (high tangents), similarly in both groups.

When neoadjuvant systemic therapy is indicated, sentinel lymph node mapping can be performed. If the axilla is clinically negative at diagnosis, mapping can be performed prior to or following systemic treatment. The advantage of performing it prior to systemic treatment lies in being able to access the information required for adjuvant treatment (radiotherapy); however, it does not affect the decision to provide neoadjuvant systemic treatment when indicated. The advantages of performing it after neoadjuvant treatment are the fact that only one surgical procedure is required; systemic treatment reduces the likelihood of lymph node positivity, particularly in biologically aggressive tumors; hence, there is less likelihood of morbidity and no delay in initiating systemic therapy. In cases of clinically positive axilla (cN + ) followed by a complete clinical response to systemic treatment (yCN0), sentinel lymph node mapping can also be performed. In this case, the false-negative rate is slightly higher than the rates prior to systemic treatment, as shown in the Z1071, sentinel neoadjuvant (SENTINA), and sentinel node biopsy following neoadjuvant chemotherapy (SN FNAC) studies (Table 3).^30^ ^31^ ^32^ Nevertheless, false-negative rates become similar when at least three sentinel lymph nodes are identified and resected. The use of a dual-tracer method or marker-clip placement on the lymph node prior to treatment improved false-negative rates in those studies. The usefulness of the random removal of lymph nodes (sampling) to reach this minimum number has yet to be confirmed scientifically. From the point of view of local control of the disease, these higher false-negative rates may have little relevance. Indeed, this criterion reflects the absence of robust studies evaluating clinical outcomes. A recent Italian study with a small number of patients, who were N+ at the beginning and experienced downstaging, failed to show any increase in axillary recurrence.^33^ Nevertheless, maximizing the false-negative rate could have played a role in the decision regarding whether to implement additional systemic treatment when the disease was identified in the breast and/or axilla.

**Table 3:** False-negative rates for sentinel lymph node mapping performed after systemic neoadjuvant therapy in clinically node-positive patients, as reported in three different studies

Study	False negative rates			
	Overall rate	≥3 sentinel lymph nodes identified	Use of immunohistochemistry	Clipped node
ACOSOG Z1071^30^	12.6%	9.1%	8.7%	6.8%
SENTINA^31^	14.2%	7.3%	–	–
SN FNAC^32^	13.3%	4.9%	8.4%*	–

Abbreviations: ACOSOG, American College of Surgeons Oncology Group; SENTINA, sentinel neoadjuvant; SN FNAC, sentinel node biopsy following neoadjuvant chemotherapy.

* Sentinel lymph node metastases of any size, including isolated tumor cells (≤ 0.2 mm), were considered positive.

Until recently, there were no randomized studies showing the advantage of performing adjuvant drug therapy following neoadjuvant chemotherapy and surgery. The CREATEx and KATHERINE studies changed this perception.^34^ ^35^ In the phase III KATHERINE trial, 1,486 patients with residual HER2 disease following neoadjuvant chemotherapy, with or without dual blockade of anti-HER2 agents, were randomized to use T-DM1 or adjuvant trastuzumab. After 3 years of follow-up, 88.3% of patients in the T-DM1 group were free of invasive disease compared with 77% in the trastuzumab group, a significant absolute difference of 11.3%, with a risk ratio of 0.50 (0.39–0.64; *p* < 0.001) and a relative reduction in recurrence of around 50%. On the other hand, the CREATEx was a randomized study involving HER2-negative women: 910 patients with residual disease following neoadjuvant chemotherapy were selected to use capecitabine or not. The study reached its primary endpoint and was stopped. In triple-negative patients, disease-free survival reached 69.8% in the women using capecitabine compared with 56.1% in the control group (0.58; 0.39–87%), a significant reduction of 42% in recurrence or death, with evidence also pointing to a benefit in overall survival (78.8% versus 70.3%), with a risk ratio of 0.52 (0.30–0.90). Therefore, in view of these new data, diagnosing residual disease in HER2-positive and triple-negative patients became crucial.

### The Future

In axillary surgery, there is an ongoing study to evaluate cN+ patients who presented as sentinel lymph node-negative following chemotherapy (ypN0).^36^ These women are being randomized to axillary dissection or conservative management. This study will provide important information on clinical outcome. Another study goes even further. Following systemic treatment, sentinel lymph node positive (ypN + ) patients are randomized to axillary dissection or radiotherapy.^37^ There are also studies being conducted to eliminate surgery to the breast and axilla.^38^ The Italian study sentinel node vs observation after axillary ultrasound (SOUND) will evaluate the omission of surgery in the axilla in the treatment of breast cancer.^39^ Finally, breast surgery is being tested in women whose clinical response after chemotherapy was excellent.^40^ After systemic treatment, evaluation including imaging and vacuum-assisted biopsy will be performed in the future to select patients for possible radiotherapy alone, without surgery.

## Conclusion

Surgical techniques have advanced from radical to conservative surgeries, thus reducing sequelae, while adjuvant and neoadjuvant therapies have contributed toward controlling the disease, both distant metastases and local recurrence. Future breast cancer therapy may progress further until surgery to the breast and axilla is completely eliminated.

## Contributions

All authors contributed equally in the conception and design, data collection, and interpretation of data. All were involved in writing the manuscript and critically reviewed its intellectual content. The final version to be published was approved by all authors.
